# Espoused implicit leadership and followership theories and emergent workplace relations: a factorial survey

**DOI:** 10.3389/fpsyg.2023.1123303

**Published:** 2023-05-10

**Authors:** Laura Hesmert, Rick Vogel

**Affiliations:** Department of Socioeconomics, Universität Hamburg, Hamburg, Germany

**Keywords:** evolutionary leadership theory, implicit followership theories, implicit leadership theories, signaling, value congruence

## Abstract

Previous research on implicit leadership and followership theories (ILTs/IFTs) and interpersonal congruence thereof has primarily focused on preexisting, vertical leader-follower dyads. This study explores interpersonal congruence of ILTs/IFTs at earliest stages of emergent workplace relations in which formal leader and follower roles are not preassigned. We suggest that ILTs/IFTs, when espoused to others, have sorting effects in the social marketplace of organizations toward adaptive workplace relations. We introduce the notion of espoused ILTs/IFTs (i.e., assumptions about leaders and followers that someone claims to have and articulates to others) and examine how congruence of self- and other-espoused ILTs/IFTs facilitates the initiation and emergence of lateral workplace relations in a ‘New Work’ design (i.e., job sharing). Results of an experimental study show that interpersonal congruence in espoused ILTs/IFTs drives attraction to a job sharing partner consistently across different types (ILTs vs. IFTs) and valences (prototypes vs. antiprototypes). While ILTs and IFTs have a similarly strong attraction effect when shared by self and other, the effect of prototype congruence is significantly larger than the effect of antiprototype congruence. The findings encourage leadership scholars to study ILTs/IFTs in a broader range of expression than hitherto and make practitioners aware of similarity biases in the formation of flexible work arrangements.

## Introduction

The notions of fit and congruence have been inherent to theory and research on implicit leadership and followership theories (ILTs/IFTs) (e.g., Veestraeten et al., 2021; Coyle and Foti, 2022). Early works in this field (Eden and Leviatan, 1975; Lord et al., 1984; Offermann et al., 1994) suggest that the fit of observed traits and behaviors of a target person with the observer’s implicit theories is an important cognitive driver of expectations of, and responses to, leadership (for reviews, see Junker and van Dick, 2014; Lord et al., 2020). Since then, scholarship has recognized that not only fit of ILTs/IFTs with perceptual leader and follower stimuli yields important attitudinal and behavioral consequences, but also congruence of implicit theories themselves. This applies to within-person congruence of ILTs/IFTs with other implicit theories, notably with leaders’ or followers’ self-conceptions (Van Quaquebeke et al., 2011a; Foti et al., 2012; Schyns et al., 2020), and for between-person congruence of ILTs/IFTs in leader-follower dyads (Riggs and Porter, 2017; Veestraeten et al., 2021; Coyle and Foti, 2022). As for the latter stream, the state-of-the-art shows that shared mental models of leadership improve leader-member exchange (Coyle and Foti, 2015; Riggs and Porter, 2017; Coyle and Foti, 2022) and, in turn, facilitate coordination through more intuitive interactions (Engle and Lord, 1997), whereas inconsistencies between implicit theories may lead to discrepancies in evaluations of the relationship quality (Van Gils et al., 2010).

Regardless the valuable insights from previous studies on ILT/IFT congruence, “the scope for future research, both in and out of the lab, in this area is vast” (Lord et al., 2020). In particular, research on ILT/IFT congruence suffers from three critical gaps that motivate the present study. First, previous research has exclusively examined ILT/IFT congruence in professional workplace relations that have already been established, while no empirical knowledge exists as to what role the consistency of ILTs/IFTs plays in the initiation and development of such relations. The preoccupation with how leadership and followership solve coordination problems in pre-existing groups at the neglect of emergent processes may be considered a more general gap in leadership studies (Acton et al., 2019; Pietraszewski, 2020). This perspective oversees the potential role of ILT/IFT congruence at earliest stages of relationship tenure, when coordinative workplace relations are built in the first place. Second, previous research has exclusively examined congruence of ILTs/IFTs among incumbents of specific roles (i.e., leaders and followers) in vertical workplace relations (Engle and Lord, 1997; Riggs and Porter, 2017; Veestraeten et al., 2021; i.e., leader-follower dyads; Coyle and Foti, 2022). While this is arguably the most likely case where ILTs/IFTs, and congruence thereof, should matter, their scope and significance in organizations may extend considerably beyond leader-follower dyads (Epitropaki et al., 2017). Research has hitherto been silent if and how ILT/IFT congruence matters when tasks are coordinated in horizontal work arrangements in which formal leadership and followership roles are not pre-assigned (Shondrick et al., 2010). Third, research on ILT/IFT congruence is imbalanced toward ILTs at the neglect of IFTs. With rare exceptions (Van Gils et al., 2010; Coyle and Foti, 2015, 2022), no prior studies have examined congruence of ILTs and IFTs simultaneously. Accordingly, the scholarly understanding of whose inter-individual congruence (i.e., ILTs’ or IFTs’) matters more remains incomplete at best.

As a consequence of these limitations, the pervasiveness of ILTs/IFTs at the workplace and the many faces of their distributed contributions to the solution of coordination problems are likely to be considerably underestimated. The purpose of this study is thus to explore inter-individual congruence of ILTs/IFTs at earlier stages of workplace relations (i.e., when such relations develop in the first place), in a different type of relation (i.e., where formal leader and follower roles are absent), and at a broader range (i.e., with ILTs and IFTs operating simultaneously) than previous research. This new and enlarged focus also invites, if not forces, to rethink the degree of salience and exposure that ILTs/IFTs may achieve and, hence, their conceptualization as ‘implicit’ theories. Although scholarship has stressed that ILTs/IFTs may reside at different levels of consciousness, repeated calls for more applications of implicit methods suggest a strong tendency toward the implicit pole of this continuum (Epitropaki et al., 2013; Lord et al., 2020). However, this perspective neglects that organizational members express their understandings and expectations of leadership and followership at various occasions and on a regular basis (Fairhurst and Connaughton, 2014). If articulated and communicated, in whatever form, the content of ILTs/IFTs becomes more tangible and travels easier within organizations than a rigorously implicit notion of ILTs/IFTs suggests. Signaling ILTs/IFTs should particularly matter at nascent stages of social relationships and groups, when members still have little or no information about each other and thus depend on social cues with whom to build professional partnerships in the social marketplace of organizations (DeRue and Ashford, 2010). From an evolutionary perspective, such signals serve an important social function (Grabo et al., 2017). Signaling attitudes and orientations that matter for the quality of exchange and the effectiveness of problem-solving, as scholarship has demonstrated for congruent ILTs/IFTs (Engle and Lord, 1997; Coyle and Foti, 2015; Riggs and Porter, 2017), reduces search costs and the risk of building or entering into ineffective relationships or groups.

We refer to ILTs/IFTs at the explicit pole of the continuum as ‘espoused’ ILTs/IFTs (for the original notion of ‘espoused theories,’ see Argyris and Schön, 1974). By espoused ILTs/IFTs, hence, we mean those assumptions about leadership and followership that someone claims to have and believes his or her behaviors are based on, in contrast to ‘theories-in-use,’ which are reflected in, and may be inferred from, actual behaviors. Accordingly, we assume that espoused ILTs/IFTs may serve as important social cues that affect the building of workplace relationships in the first place. *How does congruence between self- and other-espoused ILTs/IFTs affect the initiation of workplace relations through interpersonal attraction? How does the attraction effect vary depending on the type and valence of ILTs/IFTs?* We address these research questions in the context of ‘New Work’ designs that facilitate the self-initiated, decentralized matching of people for flexible workplace relations in organizations (Bergmann, 2019). This applied setting of self-managed and often short-lived and spontaneously emerging workplace relations is interesting to study because almost by definition, such relations are not structured along formal leader and follower roles in the first place.

Additional to our contributions specifically to the literature on congruence of implicit theories, our study responds to three broader, long-standing calls in the literature on ILTs/IFTs. First, Epitropaki et al. (2013) encourage more research at the dyadic level, where the interplay of ILTs/IFTs impacts the development of social relationships. Second, Shondrick et al. (2010) suggest moving research on ILTs/IFTs forward to more dynamic, transitory, and diffuse relationships without pre-defined roles of leader and followers. Third, Junker and van Dick (2014) call for more research on the interactions of ILTs/IFTs when they operate in concert. However, since these voids have been put on the agenda, the field has made only modest progress in filling them. We proceed along the suggested lines and help to address the gaps by examining how espoused ILTs/IFTs and their inter-individual congruence initiate largely autonomous and flexible workplace relations. The findings suggest that congruence between self- and other-espoused ILTs/IFTs indeed significantly influences the initiation of workplace relations through interpersonal attraction. On an aggregate level, this holds for different types (ILTs vs. IFTs) and valences (prototypes vs. antiprototypes) of ILTs/IFTs. On the dimensional level, however, only specific prototypes and antiprototypes matter for interpersonal attraction when shared in dyadic relationships.

## Theoretical framework and hypotheses

### Implicit leadership and followership theories congruence

ILTs/IFTs are mental representations of ideal or typical leaders and followers (Epitropaki et al., 2017; Gesang, 2022; Holm, 2023). While typical ILTs/IFTs comprise traits of average leaders or followers, ideal ILTs/IFTs are assumptions about desired or undesired traits of leaders or followers (Junker and van Dick, 2014). To the core of ILTs/IFTs are leader and follower prototypes, which comprise abstracts sets of desired traits attributed to (good) leaders or followers (such as ‘motivational,’ ‘hardworking,’ ‘team player’), whereas antiprototypes comprise negative characteristics of (bad) leaders or followers (such as ‘hostile,’ ‘arrogant,’ ‘slow’) (Offermann et al., 1994; Epitropaki and Martin, 2004; Sy, 2010). ILTs/IFTs provide organizational members with cognitive backdrops against which they perceive and evaluate stimuli from target leaders or followers (Lord et al., 1982, 1984; Epitropaki et al., 2013). It follows that members who share ILTs/IFTs will be likely to respond similarly to the same leadership and followership cues and arrive at similar definitions of social situations, including the categorization of themselves and others as leaders or followers. Previous research has established positive effects of interpersonal congruence of ILTs/IFTs, with a primary focus on congruence among leaders and followers and on the implications thereof for the quality of leader-member exchange (Engle and Lord, 1997; Van Gils et al., 2010; Coyle and Foti, 2015; Riggs and Porter, 2017; Coyle and Foti, 2022).

Evolutionary leadership theory provides theoretical substantiations of why this congruence effect occurs. From this perspective, leadership and followership are adaptive mechanisms which have evolved to ensure the stability and survival of social groups by solving problems of coordination and cooperation (Lord et al., 1984; Van Vugt, 2006; Bastardoz and Van Vugt, 2019). Shared assumptions about leadership and followership facilitate agreement among group members as to who will take these roles, what they entail, how to enact them appropriately and, hence, how to coordinate tasks. A critical issue for groups to be successful is to reach consensus about the kind and timing of collective action (Van Vugt, 2006). Acting together in unity will be easier for groups whose members share ILTs/IFTs because common beliefs foster mutual understanding and intuitive interactions (Engle and Lord, 1997), even if the content of these beliefs is not directly related to the task itself (Foss, 2001; Pietraszewski, 2020). ILT/IFT congruence will thus enhance the coordinative capacities of social groups and, ultimately, their resistance against the pressure of environmental selection at the group level. These contributions of ILTs/IFTs to the solution of coordination problems also provide an evolutionary explanation of why the content and structure of ILTs/IFTs show substantial overlaps across many societies (Den Hartog et al., 1999; Van Vugt, 2006).

It follows from this evolutionary perspective that shared assumptions about leadership and followership will not only drive effective coordination once social relationships and groups have been established, but are also likely to matter for the emergence and creation of social structures in the first place. Early sorting effects in the “social marketplace” (Pietraszewski, 2020) of organizations toward effective and against ineffective coalitions and partnerships will increase the adaptability and stability of social structures, too. If members converge early on shared cognitive blueprints of leadership and followership in emergent stages of workplace relationships, costs of failure from dissent and role conflict, including the search for a better fit, will decrease (Wellman, 2017). Thus, more efforts will be saved for the task to be coordinated.

Such sorting effects gain in importance with the proliferation of ‘New Work’ designs (Bergmann, 2019). Organizational members increasingly find themselves empowered to initiate workplace relationships that transcend traditional, more stable and hierarchical leader-follower relations, such as job sharing, peer-to-peer learning, mentoring, bottom-up projects, etc. Although many of these workplace relations are lateral rather than vertical, ILT/IFT congruence should matter in the initiation and implementation of ‘leaderless’ arrangements, for at least two reasons: First, even without formally pre-assigned leader and follower roles, associated responsibilities may emerge informally with repeated interactions – “[i]t seems that whenever a group of people come together, a leader-follower relationship naturally develops” (Van Vugt, 2006, p. 354). In addition, members may succumb to their natural tendency of self-categorizing into either a follower or leader role (Platow et al., 2003; Epitropaki et al., 2013) and thus constantly (re-)negotiate these roles and identities, which will be the less effortful and more successful if the more members share similar leadership schemas (DeRue and Ashford, 2010). Second, horizontal workplace relations are embedded in a larger environment of organizational hierarchies. Even if members in such arrangements are not leaders and followers for one another, they are likely to fulfill these roles (and may share them) toward others. Agreement on the responsibilities, tasks, and privileges associated with a leader and follower role will foster consistent attitudes and behaviors toward leaders and followers, facilitate goal-alignment and further strengthen the focus on task coordination (Drath et al., 2008; Wong et al., 2020).

### Espoused ILTs/IFTs

If it is evolutionary advantageous for social groups to emerge among members who share the same ILTs/IFTs, the question remains if and how they can know about others’ ILTs/IFTs at nascent stages of social relationships, when social information is still sparse. With regard to traditional leadership relations, previous research has provided vast evidence that various verbal and non-verbal signals fill such informational voids (Reh et al., 2017; Gerpott et al., 2018; Van Vugt and von Rueden, 2020). In the context of leadership emergence, signaling comprises all cues that, intendedly or not, flow from a potential leader to potential followers who in turn infer leadership qualities from these signals. Research has provided compelling evidence that people have enhanced abilities to recognize leadership potential in others (Van Vugt et al., 2008; Acton et al., 2019), and these abilities prevents them from following ineffective leaders. When organizational members decide whom to follow, they rely on such signals because there is often a time delay between this decision and the payoffs of followership (Van Vugt, 2006). Such situational or personal cues substitute for more relevant information that is not yet available in early stages of group formation (Bastardoz and Van Vugt, 2019).

We propose that in face of coordination challenges, ILTs/IFTs may serve as such signals, too. Although by name “implicit,” scholarship has acknowledged that ILTs/IFTs differ in the degree to which they are accessible for self-reflection and introspection (Epitropaki et al., 2013; Lord et al., 2020). Rather than operating only at subconscious levels, ILTs/IFTs reside on a broader range between implicit and explicit. Previous studies in the field have been primarily attracted to the implicit pole of the continuum, further reinforcing this tendency through repeated calls for the application of implicit methods (Epitropaki et al., 2013; Lord et al., 2020). The opposite pole, where individuals access and express their assumptions about and expectations toward leaders and followers, and where ILTs/IFTs are thus not so implicit after all, has received less attention. We refer to ILTs/IFTs at this more explicit end of the continuum as “espoused theories” (Argyris and Schön, 1974). Espoused theories are consciously held assumptions, rules, and values which people believe their behavior is based on and are able to state. Espoused ILTs/IFTs, then, are those parts of people’s assumptions and beliefs about leadership and followership which they process explicitly and articulate in organizational settings. In contrast, ILTs/IFTs-in-use operate in a preconscious fashion and drive individuals’ behaviors largely without their awareness and self-reflection (Lord et al., 1984, 2020; Foti and Lord, 1987).

We assume organizational members to espouse ILTs/IFTs on a regular basis and at various occasions. We do not argue, however, that espousing ILTs/IFTs is always a purposeful and deliberate activity, nor that it occurs in a standardized, questionnaire-ready form. Espoused ILTs/IFTs ‘in the wild’ may rather find various expressions in organizational settings. For example, feedback interventions by leaders are likely to convey images of follower prototypes if they point to desirable traits and behaviors to be developed in the future. Job applicants want to grasp what it would be like to work with a potential supervisor and will most likely learn about leader prototypes when asking for leadership cultures or styles. In informal talk and workplace gossiping, followers may criticize leaders for undesired traits and behaviors, thus reflecting and constructing leader antiprototypes. In short, espoused ILTs/IFTs are an important element of organizational discourses on leadership and followership (Fairhurst, 2008; Fairhurst and Connaughton, 2014). Through espousing in this discursive arena, organizational members make claims about themselves and position themselves relationally to others (Sacks, 1992; Fairhurst, 2008; DeRue and Ashford, 2010). Given the paramount role of leadership and followership for organizational life and beyond, it will be difficult *not* to discursively espouse ILTs/IFTs in the course of organizational membership.

As social signals, espoused ILTs/IFTs include two important bundles of information that are relevant to the evaluation of potential collaboration at the workplace. First, since ILTs/IFTs are related to self-conceptions (Van Quaquebeke et al., 2011b; Steffens et al., 2018), they will convey important information about the sender’s likely traits and behaviors in a future collaboration (Grabo et al., 2017). In particular, members express attitudinal and behavioral goals and standards when they espouse commitment to ILTs/IFTs and will be evaluated against these benchmarks afterwards. Receivers will thus make inferences about what the sender will strive for in terms of leadership and followership, which reduces uncertainty about the kind and process of future collaboration (Grabo et al., 2017). Second, and related to the previous point, espoused ILTs/IFTs signal behavioral standards that espousers are likely to apply also to others. Accordingly, espoused theories inform the receiver about the criteria against which he or she is likely to be evaluated by the sender in case of a workplace relation. For both reasons, espoused commitment to ideal ILTs/IFTs should matter more in the emergence of workplace relationships than typical ILTs/IFTs, which do not necessarily reflect attitudinal and behavioral goals and standards (Junker and van Dick, 2014).

### Congruence of espoused ILTs/IFTs

We therefore focus on ideal ILTs/IFTs and conclude that, when espoused, they have a signaling function in the social marketplace of organizations, triggering sorting effects toward effective workplace relations. Espoused ILTs/IFTs convey preferences regarding leadership and followership and thus provide valuable information with whom to coordinate tasks successfully. Following our evolutionary reasoning above, we expect a congruence effect to occur also with espoused ILTs/IFTs and already at early stages of social relationships. Members who espouse similar ILTs/IFTs will be more likely to be attracted to each other and to build workplace relations than members whose espoused ILTs/IFTs have little overlaps. In case of congruence of ILTs/IFTs, workplace relations promise to solve coordination problems more effectively, and thus to be more adaptive, because they benefit from shared priorities, higher goal alignment, better communication, and less conflict among the partners. Hence, our hypothesis is:

*H1a,b*: Congruence of self- and other-espoused (a) ILTs and (b) IFTs has a positive impact on self’s attraction to other for building a professional workplace relation.

We expect the hypothesized attraction effect to be stronger for IFT congruence than for ILT congruence. Most members in a social group will be followers, while only few will be leaders, and even fewer will be only leaders (Van Vugt et al., 2008; Bastardoz and Van Vugt, 2019; Van Vugt and von Rueden, 2020). Accordingly, more social relationships tend to unfold among followers than between leaders and followers. For the building of homogeneous and cohesive groups, espoused IFTs should therefore be more informative than espoused ILTs, and attracting individuals with similar IFTs should be more adaptive than attracting individuals with similar ILTs. Moreover, since people have self-serving definitions of social categories, ILTs/IFTs also reflect self-conceptions (Dunning, 2003; Van Quaquebeke et al., 2011b; Foti et al., 2012; Steffens et al., 2018) and signal the espouser’s motivations and identity claims. Espoused ILTs are likely to be processed as signal of a leader identity and motivation to lead, whereas espoused IFTs are signals of a follower identity and motivation to follow. Given the individual benefits of being a leader (Van Vugt et al., 2008; Bastardoz and Van Vugt, 2019), conflicts over the leadership of a group will arise among those with a strong leader identity and motivation to lead, threatening the cohesion and stability of the group. Unless the group is not too large and the task not too simple, a social structure with one leader at the top has indeed evolved as the evolutionary default of leadership (Van Vugt et al., 2008), recent considerations of collective forms of leadership notwithstanding (Contractor et al., 2012). Congruence in espousing IFTs will therefore lead to more mutual attraction than congruence in espousing ILTs, as coalitions among would-be leaders bear a higher risk of unproductive intragroup conflicts at the expense of group benefits (Van Vugt et al., 2008). Research has confirmed that group performance diminishes when too many members compete for leader status (Groysberg et al., 2011; Ronay et al., 2012). This applies all the more for relationships without formally assigned leader and follower roles because the vitality and longevity of such relationships will depend on relational qualities such as reciprocity, equality, and collegiality. Thus, we state:

*H2*: Congruence of self- and other-espoused IFTs has a stronger positive effect on self’s attraction to other for building a professional workplace relation than congruence of self- and other-espoused ILTs.

For similar reasons, we expect congruence effects to differ not only along the types of espoused ILTs/IFTs (i.e., ILTs vs. IFTs), but also along their valences (i.e., prototypes vs. antiprototypes). Prototypes are more informative signals because they express what others will be likely to expect and strive for in a workplace relation, whereas antiprototypes signal what others will avoid and disregard, leaving open what their positive goals and expectations are instead. Accordingly, espoused prototypes will reduce more uncertainty about others’ future expectations and behaviors in potential workplace relations and allow for a better informed evaluation whether group members will move in the same direction toward shared goals. Espoused prototypes are also more favorable signals in the initiation of workplace relations because they are likely to be associated with personality traits that are beneficial for an effective cooperation. For example, we expect prototype espousing to be related to a regulatory focus on promotion (i.e., a focus on gains; Higgins, 1997) because they reflect ambitions and aspirations. In contrast, espousing antiprototypes is more likely to be associated with a prevention focus (i.e., a focus on non-losses; Higgins, 1997) because antiprototypes highlight what is to be avoided. A coalition among espousers of prototypes will thus be more driven by advancement and accomplishment than a coalition among espousers of antiprototypes. This should be particularly advantageous in early stages of workplace relations, when mobilizing collective action is particularly challenging and, without a positive and shared vision for the future, prone to failure. Leadership studies have indeed corroborated that ambitious initiative-takers benefit from selection advantages in the evolution of social groups (Van Vugt, 2006). Consistent with this prediction, we assume:

*H3*: Congruence of self- and other-espoused prototypes has a stronger positive effect on self’s attraction to other for building a professional workplace relation than congruence of self- and other-espoused antiprototypes.

## Data and methods

We tested our hypotheses in an online multiplayer experiment which combined an Experimental Vignette Methodology (EVM; Aguinis and Bradley, 2014) with a collaborative task. The experiment was framed in a ‘New Work’ setting (Bergmann, 2019), more precisely in a job sharing context. Job sharing occurs when a full-time job is divided and managed by two or more employees who are mutually responsible for the position (Krone-Germann and Guénette, 2016). This self-initiated and self-organized work arrangements is an interesting setting for leadership and followership studies beyond traditional leader-follower relations, as has repeatedly been called for in the literature (Shondrick et al., 2010; Bastardoz and Van Vugt, 2019).

### Participants

A total of 308 participants took part in the experiment. We recruited participants from two sources: First, we sampled from a pool of respondents managed by a large public university’s internal experimental laboratory services, which consists of both undergraduate and graduate social sciences students as well as employees. Second, since the target group of job sharers is difficult to reach, we commissioned a panel provider to specifically invite employees with job sharing experience. All participants received a monetary compensation in return for their participation in the experiment. We excluded participants with extremely long or short response rates (+/− 2 SD), resulting in a final sample of *N* = 304. Women made up 55.2% of the sample and 58.2% of all participants were students. On average, participants were about 37.5 years old (SD = 15.7). Of all participants, 93.4% were currently employed, 46.1% indicated to have experience in job sharing, and 55.2% had leadership experience.

### Procedure

**Figure 1:**
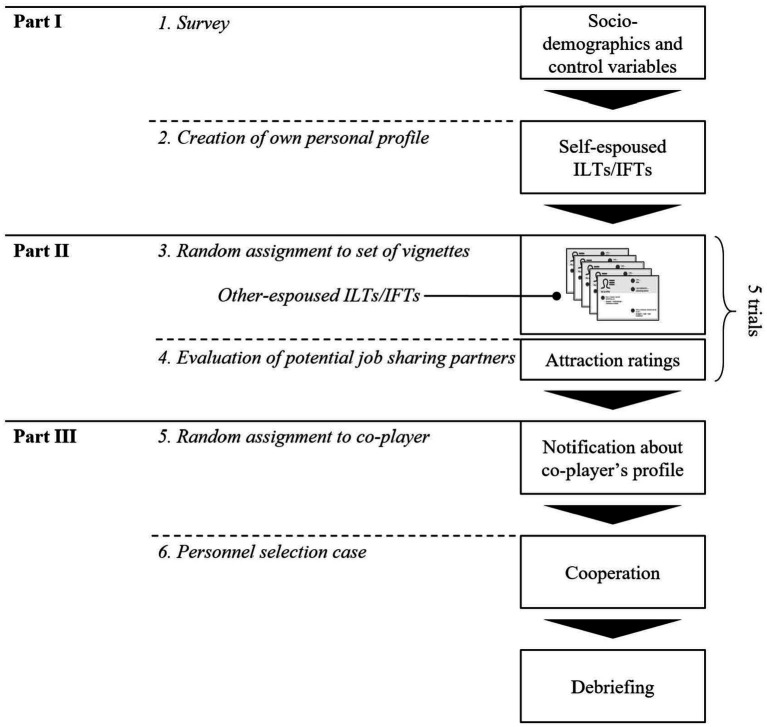
Schematic overview of experimental procedure.

A schematic overview of the experimental procedure is illustrated by Figure 1. A detailed overview of the experimental procedure, including instructions and used material, is provided by Appendix A. We set up a total of 27 sessions, in each of which 10 to 15 people participated simultaneously. One week prior to the data collection, the panel data provider distributed an invitation link *via* its message system. By clicking on the link, individuals who were interested in participating were led to an online appointment management system where they could sign up for a session. Two hours prior to the scheduled session, participants received a single-use access link to the online experimental platform. Participants’ anonymity was guaranteed throughout the whole procedure.

### Part I–demographics and self-espoused ILTs/IFTs

After signing the consent, privacy and confidentiality forms, participants completed the first part of the experiment. In Step 1, they provided their demographics, precisely their age, work status, job sharing, and leadership experience. In Step 2, participants were instructed that they participate in a multiplayer game, in which teams of two competed against each other in a collaborative task associated with job sharing. They were informed that the winning team would be awarded a price money of €20. Participants were told that team couples were matched based on their personal preferences regarding leadership and followership. Therefore, each player had to first create their own personal profile with personal statements on ideal leaders and followers. For this purpose, participants were presented a list of 12 statements, each of which represented an ILT/IFT prototype or antiprototype. The statements were introduced with the phrase “*How a leader [follower] should definitely be…”* for prototypes or “*How a leader [follower] should not be at all…*” for antiprototypes, respectively, and completed by three attributes comprising the (anti-)prototype (e.g., “*dynamic, motivational, confidence builder*” for *Inspirational*; Table 1). Via clicking on check boxes next to the statements, participants could select up to two statements to appear in their personal profile. They were not given any constraints regarding the combination of ILTs/IFTs or prototypes/antiprototypes. As a result of their selection, participants created a profile that contained their self-espoused ILTs/IFTs. The profiles resembled real-life examples from an online platform for job sharing^1^ and were pre-tested for face validity with registered users of the platform. A sample profile is illustrated in Appendix B.

**Table 1:** Leader and follower prototypes and antiprototypes.

Type and valence	Dimension	Items
Leader prototype*^a^*	Inspirational	*Dynamic, motivational, confidence builder*
	Performance Orientation	*Improvement oriented, excellence oriented, performance oriented*
	Team Integrator	*Informed, team builder, integrative*
Leader antiprototype*^a^*	Self-Centered	*Self–interested, loner, asocial*
	Face Saver	*Indirect, avoids negatives, evasive*
	Malevolent	*Vindictive, hostile, irritable*
Follower prototype*^b^*	Industry	*Hardworking, productive, goes above and beyond*
	Enthusiasm	*Excited, outgoing, happy*
	Good Citizen	*Loyal, reliable, team player*
Follower antiprototype*^b^*	Conformity	*Easily influenced, follows trends, soft spoken*
	Insubordination	*Arrogant, rude, bad–tempered*
	Incompetence	*Uneducated, slow, inexperienced*

^a^Brodbeck et al. (2000).

^b^Sy (2010).

### Part II–experimental vignette methodology

In Step 3, participants were informed that they would next be asked to express their preferences regarding a job sharing partner. Participants were reminded that their ratings would be used by the matching algorithm to match team members afterwards. In the following, participants were randomly assigned to a set of five personal profiles (i.e., the vignettes) in randomized order, each of which represented the personal profile of a potential job sharing partner and their espoused ILTs/IFTs. In order to arrive at an even distribution of these (anti-)prototypes, the presented profiles were not those created by other participants in the previous step but computer-generated. Twelve dimensions given (i.e., 6 ILT and 6 IFT dimensions; Table 1), 12 × 12 = 144 combinations were possible. However, since the order of presentation did not matter and espousing the same dimension twice did not make sense, we reduced the vignette universe to the upper or lower half of the matrix, i.e., to 66 vignettes. We added 12 vignettes including only one dimension and leaving the other blank in order to obtain ‘empty’ reference categories for the data analysis. We furthermore varied the gender of the job sharing candidates randomly across the vignettes, with half of the profiles supposedly created by men and the other half by women. In Step 4, participants indicated the extent to which they wished to engage in the job sharing task with the respective player. This step was repeated for each of the presented profiles.

### Part III–cooperative decision task

Upon completion of the EVM, participants saw their assigned job sharer’s personal profile on a screen, supported by the notification that they would complete the final part of the experiment, i.e., the job sharing task, with them (Step 5). The task (Step 6) was based on a personnel selection case, inspired by a hidden profile paradigm (Schulz-Hardt and Mojzisch, 2012). Since the purpose of this task was to increase the consequentiality of participants’ ratings in the first and second part of the experiment, we do not go into detail here and refer to Appendix C instead. In the debriefing of the experiment, participants were informed that the matching of co-players was, in fact, random (i.e., independent from their ratings of the co-players’ profiles) and that the presented profile of the job sharer was random, too (i.e., not created by the assigned co-player). The design of the experiment was approved by the research ethics committee of the authors’ university.

### Measures

#### Interpersonal attraction

We considered the initiation of a professional workplace relation as an instance of interpersonal attraction. Accordingly, we operationalized our dependent variable as the extent to which participants wanted to share a job with the co-players presented in the profile rating task (Step 4). The item was “*Please indicate the extent to which you would like to engage in job sharing with this person.*” Responses on a Likert-type scale ranged from 1 = “*not at all*” to 7 = “*very much.*”

#### Congruence between self- and other-espoused ILTs/IFTs

We operationalized congruence between self- and other-espoused ILTs/IFTs as the match between self’s and other’s espoused ILTs/IFTs. We constructed dummy variables that indicated if the statements in self’s profile (created in Step 2) and in other’s profile (presented in Step 3) had overlaps or not. The value of each congruence measure was 1 if self and other espoused the same dimension of ILTs/IFTs and 0 otherwise. Beyond the dimensional level of ILTs/IFTs, we aggregated the congruence measures for the type and valence of ILTs/IFTs (i.e., ILT, IFT, prototype, antiprototype) and all combinations thereof (i.e., leader prototype, leader antiprototype, follower prototype, follower antiprototype). We retrieved the ILT items (Table 1) from the Global Leadership and Organizational Behavior (GLOBE) leadership scales for ideal and counter-ideal ILTs (Den Hartog et al., 1999). We focused on the three dimensions with the highest (i.e., prototypes) and lowest prototypicality ratings (i.e., antiprototypes) in the German regional cluster (Brodbeck et al., 2000). For IFTs, we used Sy’s full six-dimensional scale (Sy, 2010).

#### Control variables

We controlled for several socio-demographic characteristics of the respondents. First, empirical evidence supporting the socioemotional selectivity theory (Carstensen, 1992) has demonstrated that with higher age, people selectively reduce their social interactions and, as a consequence, become less open to building new relationships. In addition, there is ample evidence that work-related attitudes change as function of chronological age (Ng and Feldman, 2010). These results suggest that both the general propensity to engage in job sharing and the attitudes driving the attraction to a potential job sharing partner may differ between younger and older participants. Second, we control for gender, since gender biases are persistent in ILT- and IFT-related categorization processes (e.g., Braun et al., 2017; Offermann and Coats, 2018) and thus may confound the attraction ratings of job sharing partners. Third, employment status may be relevant because unemployed people may perceive a higher pressure to find a partner for a new job than employed people. Fourth, the assessment of candidates may depend on respondents’ job sharing experience. Participants that made positive or negative experiences with job sharing partners in the past may modify their evaluation criteria. Fifth, people’s attitudes about leadership and followership are likely to change when they adopt a leadership role themselves (Epitropaki and Martin, 2004). This may, in turn, affect the relevance of a candidate’s espoused commitment to certain ILTs and IFTs for his or her attractiveness as a job sharing partner. Therefore, we control for leadership experience.

#### Analysis strategy

To account for the hierarchical data structure (i.e., vignettes clustered within respondents), we applied multi-level regression analysis, or hierarchical linear modeling, and estimated random intercept models with independent variables at the within-level (level 1) and the control variables at the within- and between-levels (level 2). All models were estimated using RStudio (R Core Team, 2015).

## Results

Means, standard deviations, and intercorrelations of the study variables on both levels of analysis are displayed in Tables 2 3. Table 4 presents the results of the multi-level regression analyzes with attraction to a job sharing partner as the dependent variable. The intraclass correlation coefficient (ICC) in the null model (not reported) is 0.207. Thus, about 21% of the variance in the dependent variable is due to random effects at the level of respondents rather than at the level of vignettes. Observations are thus indeed not independent.

**Table 2:** Descriptives and Intercorrelations of Level 2-variables (*N* = 304).

Variable	*M*	SD	1	2	3	4	5
1. Gender (1 = male)	0.75	6.77	−0.02				
2. Age	37.52	15.70	0.06^**^	0.14^**^			
3. Work Status (1 = employed)	1.33	0.59	0.07^**^	0.08^**^	0.27^**^		
4. Leadership Experience (1 = yes)	0.45	0.50	−0.05	0.35^**^	0.45^**^	0.21^**^	
5. Job Sharing Experience (1 = yes)	0.69	0.46	−0.27^**^	0.01^**^	0.07^**^	0.22^**^	0.39^**^

Except for age, point-biserial correlation coefficients are reported.

**p < 0.001, ^**^p < 0.01, ^*^p < 0.05, two–tailed.

**Table 3:** Intercorrelations of Level 1-variables (*N* = 1,520).

Variable	1	2	3	4	5	6	7	8	9	10	11	12	13	14	15	16	17
1. Interpersonal attraction																	
2. ILT	0.04																
3. IFT	−0.02	−0.40^**^															
4. Prototype	0.18^**^	0.08^**^	−0.02														
5. Antiprototype	−0.11^**^	−0.06^*^	0.04	−0.37^**^													
6. Leader prototype	0.09^**^	0.56^**^	−0.34^**^	0.51^**^	−0.39^**^												
7. Leader antiprototype	−0.02	0.56^**^	−0.38^**^	−0.29^**^	0.46^**^	−0.18^**^											
8. Follower prototype	0.13^**^	−0.32^**^	0.49^**^	0.56^**^	−0.34^**^	−0.23^**^	−0.27^**^										
9. Follower antiprototype	−0.12^**^	−0.44^**^	0.54^**^	−0.38^**^	0.54^**^	−0.34^**^	−0.29^**^	−0.23^**^									
10. ILT congruence	0.09^**^	0.17^**^	−0.17^**^	0.05	−0.06^*^	0.16^**^	0.06^*^	−0.10^**^	−0.11^**^								
11. IFT congruence	0.15^**^	−0.20^**^	0.19^**^	0.07^**^	−0.06^*^	−0.11^**^	−0.16^**^	0.21^**^	0.05^*^	−0.06^*^							
12. Prototype congruence	0.16^**^	−0.03	0.02	0.22^**^	−0.20^**^	0.09^**^	−0.13^**^	0.18^**^	−0.12^**^	0.49^**^	0.58^**^						
13. Antiprototype congruence	0.07^*^	−0.02	0.02	−0.16^**^	0.15^**^	−0.09^**^	0.07^**^	−0.11^**^	0.12^**^	0.37^**^	0.35^**^	−0.07^**^					
14. Leader prototype congruence	0.10^**^	0.13^**^	−0.14^**^	0.13^**^	−0.15^**^	0.25^**^	−0.07^**^	−0.08^**^	−0.10^**^	0.78^**^	−0.04	0.65^**^	−0.04				
15. Leader antiprototype congruence	0.03	0.10^**^	−0.08^**^	−0.10^**^	0.10^**^	−0.07^**^	0.20^**^	−0.07^**^	−0.04	0.57^**^	−0.06^*^	−0.06^*^	0.68^**^	−0.03			
16. Follower prototype congruence	0.13^**^	−0.16^**^	0.16^**^	0.17^**^	−0.13^**^	−0.09^**^	−0.12^**^	0.31^**^	−0.07^**^	−0.05	0.81^**^	0.73^**^	−0.06^*^	−0.02	−0.05		
17. Follower antiprototype congruence	0.06^*^	−0.12^**^	0.10^**^	−0.12^**^	0.10^**^	−0.06^*^	−0.10^**^	−0.09^**^	0.19^**^	−0.04	0.54^**^	−0.05	0.71^**^	−0.03	−0.03	−0.04	
18. Other’s gender (1 = male)	−0.02	−0.10^**^	0.12^**^	0.19^**^	−0.23^**^	0.41^**^	−0.50^**^	−0.03	0.11^**^	0.04	0.05^*^	0.09^**^	−0.04	0.15^**^	−0.14^**^	0.00	0.09^**^

*** *p* < 0.001, **p < 0.01, *p < 0.05, two–tailed.

**Table 4:** Multi-level regression; DV, attraction to job sharing partner.

	Model I				Model II				Model III			
Predictors	*b*	SE	CI_95%_	*p*	*b*	SE	CI_95%_	*p*	*b*	SE	CI_95%_	*p*
(Intercept)	4.13	0.13	3.88–4.39	**<0.001**	4.14	0.13	3.88–4.40	**<0.001**	4.13	0.13	3.87–4.39	**<0.001**
Interpersonal congruence
IFT congruence (d)	1.14^***^	0.14	0.87–1.41	**<0.001**								
Prototype congruence (d)					1.33^***^	0.13	1.08–1.58	**<0.001**				
Antiprototype congruence (d)					0.64^***^	0.17	0.30–0.98	**<0.001**				
Leader prototype congruence (d)									1.21^**^	0.18	0.86–1.57	**<0.001**
Leader antiprototype congruence (d)									0.73^**^	0.24	0.25–1.21	**0.003**
Follower prototype congruence (d)									1.38^***^	0.16	1.07–1.70	**<0.001**
Follower antiprototype congruence (d)									0.55^*^	0.24	0.09–1.02	**0.021**
Controls
Other’s gender (d; 1 = male)	−0.10	0.08	−0.26–0.05	0.190	−0.12	0.08	−0.28–0.03	0.112	−0.11	0.08	−0.27–0.04	0.151
Self’s gender (d; 1 = male)	−0.07	0.13	−0.32–0.18	0.580	−0.07	0.13	−0.33–0.18	0.580	−0.07	0.13	−0.33–0.18	0.579
Self’s age	−0.01	0.01	−0.02 − 0.01	0.352	–0.01	0.01	−0.02 − 0.01	0.360	–0.01	0.01	−0.02–0.01	0.371
Self’s employment (d; 1 = male)	0.24	0.21	−0.16–0.64	0.244	0.25	0.21	−0.16–0.66	0.233	0.24	0.21	−0.17–0.65	0.246
Self’s job sharing experience (d; 1 = yes)	0.50^***^	0.15	0.22–0.79	**<0.001**	0.49^***^	0.15	0.20–0.78	**<0.001**	0.49^***^	0.15	0.20–0.78	**<0.001**
Self’s leadership experience (d; 1 = yes)	0.11	0.16	−0.20–0.42	0.487	0.13	0.16	−0.19–0.45	0.421	0.14	0.16	−0.18–0.45	0.401
*σ* ^2^		2.09					2.07				2.06	
*τ*_00_		0.64					0.67				0.67	
ICC		0.23					0.24				0.25	
*N*_Level 1/Level 2_		1,520/304					1,520/304				1,520/304	
Marginal *R*^2^/conditional *R*^2^		0.073/0.289					0.078/0.304				0.080/0.307	
AIC		5758.769					575.326				5751.404	

^***^p < 0.001, ^**^p < 0.01, ^*^p < 0.05. Significant effects with bold *p*-values.

### Hypothesis testing

Hypothesis 1a states that self’s attraction to other is contingent on congruence in espoused ILTs. We find support for this effect in Model I (Table 4), as the coefficient for ILT congruence is positive and significant (Model I; *b* = 1.03, *p* < 0.001). This dummy-coded predictor indicates if there was a match between self and other in at least one of the six ILT dimensions (Table 2), regardless of the content of the congruent dimension. Hypothesis 1b states that espoused IFTs will affect interpersonal attraction, too. The findings support this assumption (*b* = 1.14, *p* < 0.001). We thus accept both Hypothesis 1a and 1b.

Hypothesis 2 predicts that self’s attraction to other is more strongly associated with IFT congruence than with ILT congruence. While the regression weight of IFT congruence (*b* = 1.14, *p* < 0.001) is indeed larger than the weight of ILT congruence (*b* = 1.03, *p* < 0.001), the 95%-confidence intervals reveal that they overlap by more than 25%. According to Cumming (2009), this allows for the conclusion that there is no statistically reliable difference in the regression coefficients. We conclude that the effects of IFT and ILT congruence, while highly significant, do not differ substantially, thus rejecting Hypothesis 2.

Hypothesis 3 predicts that the effect of congruence on attraction is stronger for prototypical than for antiprototypical dimensions of espoused ILTs/IFTs. Analogous to Model I, the predictors in Model II indicate if there was a match between self and other in at least one prototypical or antiprototypical ILTs/IFTs dimension, respectively. Both prototype (*b* = 1.33, *p* < 0.001) and antiprototype congruence (*b* = 0.64, *p* < 0.001) have positive and significant effects on interpersonal attraction. The confidence intervals for the model estimates of prototype and antiprototype congruence do not overlap, thus pointing to a significant difference between the estimates (Cumming, 2009). To further corroborate this inference, we compared a model assuming parallel slopes for both predictors with a model including an interaction term for the predictors, i.e., crossing slopes (West et al., 2014). The model comparison revealed that we could not reject the null hypothesis of equal slopes for ILT and IFT congruence. This indicates that the model estimates for prototype and antiprototype fit are indeed significantly different. We thus confirm Hypothesis 3.

As robustness check, we re-estimated all models without controls but found results only marginally changed. All additional models were presented to the reviewers but are not included in this article in order to save space.

### Post-hoc analyses

Beyond hypothesis testing, we delved deeper into the hypothesized congruence effects in three post-hoc analyses. First, we were interested in the effect of congruence at the next lower level of aggregation, where type and valence of ILTs/IFTs combine into four predictors (i.e., ILT/IFT (anti-)prototypes). All four combinations have a significant positive impact on attraction, which is in line with our hypotheses (Model III, Table 4). Replicating the pattern that emerged in Model I, the 95%-confidence intervals and model comparisons reveal that the relative impact of congruence in leader prototypes (*b* = 1.21, *p* < 0.001) on attraction does not differ significantly from the impact of congruence in leader antiprototypes (*b* = 0.73, *p* = 0.003). However, congruence in follower prototypes (*b* = 1.38, *p* < 0.001) has a significantly larger influence on attraction than follower antiprototype congruence (*b* = 0.55, *p* = 0.021).

**Figure 2:**
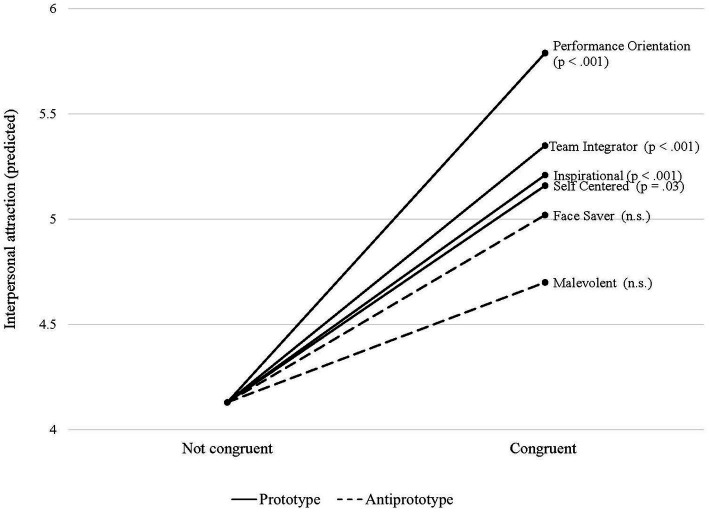
Marginal effects of ILT dimensions.

Second, we delved into the dimensional level of espoused ILTs/IFTs and examined the attraction effects of congruence in the 12 individual dimensions. Given this high number of predictors, we do not report the estimated model but rather illustrate marginal effects for all dimensions (Figures 2 3). For each dimension, we predicted the marginal means. As the figure shows, only some of the separate ILTs/IFTs matter for interpersonal attraction when shared among self and other.

**Figure 3:**
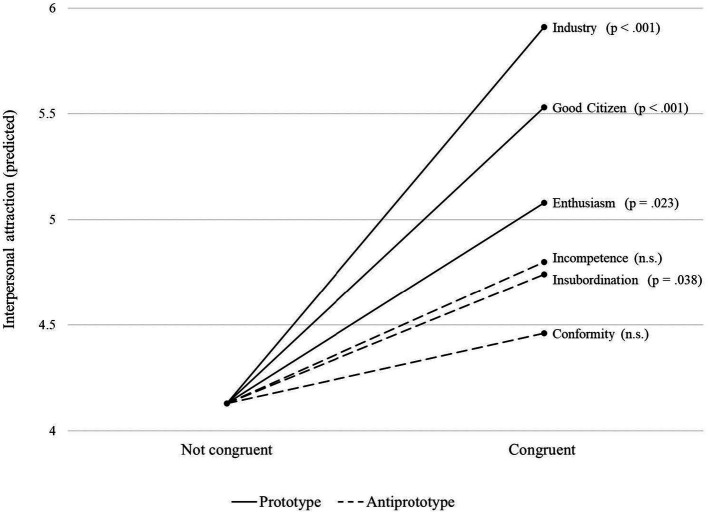
Marginal effects of IFT dimensions.

Third, we spent attention to potential plateauing effects at increasing levels of congruence. We recoded the independent variables as count variables corresponding to the numbers of matches of self- and other-espoused ILTs/IFTs. As the profiles included a maximum of two (anti-)prototypes, the values were 0 (for no match), 1 (for one match) or 2 (for two matches). We subjected these variables to three hierarchical regression models mirroring the specifications in Table 4. In a pairwise contrast analysis, we tested the statistical difference between the three congruence levels of each predictor to inspect whether attraction ratings vary with different degrees of congruence. The results (Appendix C) reveal that there is no plateauing effect for congruence of prototypes in general, as increases both from 0 to 1 and from 1 to 2 matches add substantially and significantly to the attraction effect. In contrast, the effects of leader prototype congruence more specifically and antiprototype congruence in general plateau after one match. We should note that we cannot make similar inferences for all types of congruence because participants did not self-espouse ILTs/IFTs evenly across all available categories. Accordingly, some combinations of self- and other-espoused ILTs/IFTs lack a sufficient number of observations for this kind of analysis.

## Discussion and conclusion

Professional workplace relations among organizational members who share ILTs/IFTs foster the coordination of tasks through higher goal alignment and better understanding of how to get things done (Engle and Lord, 1997). Evolutionary theory suggests that it will be even more advantageous and adaptive for organizations if members with similar ILTs/IFTs do not find themselves in workplace relations as a matter of coincidence but as the result of interpersonal attraction already in the emergence of such relations, when social information is still sparse and clear leadership and followership structures are absent (Wellman, 2017; Pietraszewski, 2020). Such sorting effects in the social marketplace of organizations become increasingly important with the proliferation of self-initiated and self-managed work designs beyond vertical leader-follower dyads (Bergmann, 2019). We propose that ILTs/IFTs, when espoused in the discursive arena of organizations, serve as verbal signals that fill informational gaps and trigger the emergence of such work arrangements, thus facilitating goal alignment, saving costs of failure, and promoting successful collaboration. The results broadly confirm that congruence of self- and other-espoused ILTs/IFTs has early sorting effects through interpersonal attraction for building workplace relations.

The attraction effect of interpersonal congruence holds for both ILTs and IFTs, in similar strengths. While we hypothesized that similarity in both ILTs and IFTs will have a positive impact on interpersonal attraction, we expected the effects of IFT congruence to be stronger than those of ILT congruence. Previous scholarship is largely silent on this issue, such that our study presents novel evidence. The findings do not confirm a priority of IFTs over ILTs, nor the other way round. This may point to the duality of leadership and followership, which have coevolved as reproductive strategies (Meindl and Ehrlich, 1987; Van Vugt, 2006). While congruence in individuals’ attitudes toward followership is adaptive to build a functional base of a leader-follower hierarchy, it also requires uniform support and endorsement from all group members for leaders at the top of this hierarchy (Van Knippenberg et al., 2004). Even if designed as ‘leaderless,’ horizontal workplace relations are embedded in such hierarchies, with involved members being followers of leaders and leaders of followers. Pre-selecting group members with congruent ILTs will thus make a unique contribution to coordination efficiency, beyond the congruence effect of IFTs. Agreement on ILTs is all the more important when leadership roles and responsibilities toward others are shared and fluctuate over time, as is frequently the case in job sharing dyads (Krone-Germann and Guénette, 2016). Finally, horizontal workplace relations may not be so leaderless after all, as leadership may emerge informally among the involved members. Thus, considering both ILTs and IFTs may prepare for the recursive and dynamic negotiation process of leadership emergence (DeRue and Ashford, 2010; Acton et al., 2019).

The attraction effect of interpersonal congruence also holds for both prototypes and antiprototypes. This finding provides supporting evidence for a two-force perspective, according to which relational outcomes are contingent on congruence of both ideal and counter-ideal values and attitudes (Van Quaquebeke et al., 2010; Graf et al., 2011; Schuh et al., 2018). However, and still consistent with these findings, congruence in prototypes explains interpersonal attraction to substantially larger extents. We thus find our evolutionary reasoning confirmed, according to which espoused antiprototypes are less informative cues in the initiation of social relationships than prototypes. Accordingly, congruence in antiprototypes may be more a ‘nice-to-have’ than a ‘must-have.’ This explanation also resonates with the literature on attribute framing (Levin et al., 1998), which posits that negative framings of signals (i.e., espoused antiprototypes) result in less favorable ratings than positive framings (i.e., espoused prototypes). As consequence of valence-based associative processing, an option appears less attractive and is rated less favorably when labeled in negative rather than positive terms, leading to compatibility between stimulus and response. Partnering with members who share the same favorable attitudes is thus likely to be perceived as more attractive and adaptive than partnerships among members who share less favorable attitudes.

The paramount role of prototype congruence is also reflected in two post-hoc findings that provide new insights, compared to previous research: First, the attraction effect does not plateau with increasing levels of congruence. However, we admit that our operationalizations and data did not allow for more fine-grained analyses of plateauing effects, which we thus leave for future research. Second, all prototypical dimensions increase interpersonal attraction in significant margins if shared by self and other, whereas congruence in only two antiprototypical dimensions exhibits significant marginal effects: self-centered (ILT) and insubordination (IFT). Arguably, these dimensions are interrelated because they reflect a lack of social skills of leaders (‘loner,’ ‘asocial’) and followers (‘arrogant,’ ‘rude’), respectively, and may cast doubts on someone’s willingness to comply with group norms. Prospective job sharers should have a strong avoidance orientation toward these traits, as they are likely to affect the quality and adaptability of the job sharing partnership. Instead, the search for a collaboration partner should rather be guided by criteria such as agreeableness, sportsmanship, and teamwork skills. This is suggested by our finding that congruence in the prototypes of *industry* (i.e., ‘hardworking,’ ‘productive,’ ‘goes above and beyond’) and *good citizen* (i.e., ‘loyal,’ ‘reliable,’ ‘team player’) have the largest positive effects on interpersonal attraction.

Our findings contribute not only to the academic literature but also yield some practical implications for ‘New Work’ designs in general and job sharing arrangements more particularly. Our results show that in self-guided matchmaking processes, job sharers are likely to form couples with someone who is ‘on the same page’ regarding leadership and followership. This homophily effect has ambiguous consequences. On the one hand, and consistent with our evolutionary reasoning, similar partners are likely to coordinate tasks more effectively, thus building more adaptive and trusted relationships. On the other hand, research on team composition shows that cognitive diversity facilitates creative thinking and problem-solving under high task uncertainty (e.g., Schilpzand and Martins, 2010; Liao and Long, 2016). Leaders and human resource professionals thus have to balance conflicting demands when they decide how, and if at all, they intervene in the matchmaking process. Depending on job and tasks characteristics, they may consider counter-balancing members’ strive for supplementary fit and foster complementary fit instead.

### Limitations

As any research, our study is subject to some limitations. First, although experimental designs come along with a number of advantages, they raise concerns about external validity. We have made several arrangements to increase external validity, starting with the overall choice of an EVM (Aguinis and Bradley, 2014). However, it still remains unclear how our findings generalize to judgments and decisions in real-life contexts. In particular, we manipulated espoused ILTs/IFTs in the stylized fashion of questionnaire items (Den Hartog et al., 1999; Sy, 2010), whereas espousing in the ‘wilderness’ of organizations is likely to occur in various, less standardized forms. Second, given long-standing debates about the measurement of fit (Edwards, 2008), our binary measure of congruence is simple. This is a consequence of our experimental stimuli, which did not include different grades of approval or rejection of ILTs/IFTs for reasons of simplicity and unambiguity. Future research could move forward to more nuanced signals and examine gradual levels of espousing ILTs/IFTs, paving the way to the application of more advanced congruence measures (e.g., Riggs and Porter, 2017). Third, and related to the previous point, we examined congruence only at the top of the respondents’ preference hierarchies because they were exposed to only one or two ILTs/IFTs in each candidate profile and created their own profiles according to the same design. Future research could broaden the scope to congruence in ILTs/IFTs at lower levels of (un-)desirability. Fourth, job sharers are a specific population for which almost no data is available. While a substantial proportion of our respondents stated to have job sharing experience, we could not test this subsample for representativeness.

### Research outlook

Besides these limitations, our focus on earliest stages of horizontal workplace relations comes along with a novel perspective on the varying degree of consciousness, articulation, and communication that ILTs/IFTs may have in organizational life. Our distinction between espoused ILTs/IFTs and ILTs/IFTs-in-use, although a conceptual simplification of what is likely to be an empirical continuum, offers new avenues for further research. The tension of conceptualizing ILTs/IFTs as implicit while asking respondents for explicit statements, notably in the plethora of survey studies, is inherent to previous scholarship (Lord et al., 2020). The notion of espoused ILTs/IFTs suggests that researchers may consider explicitly stated and claimed theories about leadership and followership as a relevant phenomenon worth studying in itself, rather than as source of measurement errors.

Our focus was on interpersonal congruence of espoused ILTs/IFTs, but the intrapersonal relationship of espoused ILTs/IFTs and ILTs/IFTs-in-use remains to be explored. For example, how does espousing of ILTs/IFTs through verbal expression affect the semantic memory where ILTs/IFTs-in-use reside? To what extent do they converge, and what are the antecedents and consequences of this convergence or divergence? Do individuals engage in comparisons between their espoused theories and theories-in-use, and to what extent are they aware of potential discrepancies? These questions shed light on the increasingly discussed, however largely unexplored role of ILTs/IFTs in context-specific leader and follower identity constructions (Lord et al., 2020). Epitropaki et al. (2017) suggest that if people perceive a match between their own ILTs/IFTs and their behavior, they will more likely identify as a leader and develop motivation to lead. However, the few studies which have examined the actual consequences of self-to-prototype comparisons empirically (Foti et al., 2012; Guillen et al., 2015) have largely neglected the role of relational signals, e.g., a potential partner’s self-disclosure of identity claims, behavioral standards, and interactional goals encoded in their espoused ILTs/IFTs. Expanding on our study, future research is invited to explore espoused ILTs/IFTs as a connecting piece in the puzzle of leadership categorization and identity processes. Exploring these and other relationships across different contexts and over time sets a rich agenda.

## Data availability statement

The raw data supporting the conclusions of this article will be made available by the authors upon reasonable request, without undue reservation.

## Ethics statement

Ethical review and approval was not required for the study on human participants in accordance with the local legislation and institutional requirements. The patients/participants provided their written informed consent to participate in this study.

## Author contributions

LH and RV contributed to the conception and design of the study, performed the statistical analysis and wrote this manuscript. All authors contributed to the article and approved the submitted version.

## Conflict of interest

The authors declare that the research was conducted in the absence of any commercial or financial relationships that could be construed as a potential conflict of interest.

## Publisher’s note

All claims expressed in this article are solely those of the authors and do not necessarily represent those of their affiliated organizations, or those of the publisher, the editors and the reviewers. Any product that may be evaluated in this article, or claim that may be made by its manufacturer, is not guaranteed or endorsed by the publisher.

## Footnotes

^1^ https://app.tandemploy.com/en/home
