# Supplementary material for: Espoused implicit leadership and followership theories and emergent workplace relations: a factorial survey
Source: Front Psychol. 2023 May 10;14:1123303. doi: 10.3389/fpsyg.2023.1123303 (PMC10206136; doi:10.3389/fpsyg.2023.1123303)
Supplement: Supplementary file 1 [file Data_Sheet_1.pdf]

Appendix

Appendix A. Detailed Experimental Procedure

Page	Title	Text/Image on Screen
**Part I Survey and Personal Profile**		
1.1	Welcome and introduction	Dear participant, Thank you for taking the time to participate in our study! This study explores how people cooperate in job sharing relationships. Before we begin, please make yourself familiar with our privacy and confidentiality regulations and sign the consent form on the next page.
1.2	Demographics and Controls	Great, Thank you. Before we start with the main part of the study, we kindly ask you to provide some information about yourself.<br>1. *What gender do you identify with?*<br>a. *Male*<br>b. *Female*<br>c. *Diverse*<br>2. *How old are you?*<br>a. *[Please indicate here]*<br>3. *Please indicate your current employment status!*<br>a. *Employed*<br>b. *Currently unemployed*<br>4. *Are you currently or have you ever been in a leadership position? (Note: You are/were in a leadership position if you are/have been supervising staff).*<br>a. *Yes, am currently in a leadership position*<br>b. *Yes, have been in a leadership position, but not currently*<br>c. *No*<br>5. *Do you have experience in job sharing with other workers (job sharing)*?*<br>a. *Yes*<br>b. *No*<br>* *Job sharing refers to the practice of dividing the tasks, responsibilities and the pay of one job between two people who work at different times during the day or week.*
1.3	Overview and general instruction	Wonderful. We will now begin with the main study. In the following, you and 5 other people will take part in a multiplayer game in which teams of two will compete against each other in a job sharing task. To form teams, couples will be drawn from the group of people who participate at the moment and will be matched (i.e., assigned to each other) to form a team. The best performing team will receive an additional payout of 20 € (10 € per player).<br>The game consists of three rounds. In round 1, we ask you to create a personal profile of yourself. In round 2, you will have the opportunity to express your preferences regarding a job sharing partner for the task in round 3. Based on this information, an algorithm matches you with a partner at the end of round 2.<br>In round 3, you will work on a task together with the partner assigned to you. We will explain the details of the task later in the experiment.

<table>
<tr><td>1.4</td><td>Instruction personal profile</td><td>Let's begin with the first round. We would like to ask you to create a personal profile of yourself. The profile you create will be presented to all players in round 2, together with your co-players’ profiles. In round 2, every player will be able to select their preferred job sharing partners for round 3 from all presented profiles.</td></tr>
<tr><td>1.5</td><td>Creation of personal profile</td><td> Your personal profile will contain your gender and personal preferences regarding leaders and followers. To create a profile, please select a maximum of two statements from the statements below. There are no restrictions to your selection: Statements can be selected across categories (i.e., the grey boxes) and also both stem from the same category. Once you hit “continue”, your profile will be created automatically based on your selection.
Please note: The profile will be completely anonymized and except from your gender, your personal and demographic data will NOT be published.
<table>
<tr><th colspan="4">PLEASE SELECT A MAXIMUM OF TWO FROM THE STATEMENTS BELOW!</th></tr>
<tr><th colspan="2">How a <u>leader</u> should definitely be ...</th><th colspan="2">How a <u>leader</u> should not be at all ...</th></tr>
<tr><td>☐</td><td>dynamic + motivational + confidence builder</td><td>☐</td><td>indirect + avoids negatives + evasive</td></tr>
<tr><td>☐</td><td>improvement- + excellence- + performance oriented</td><td>☐</td><td>vindictive + hostile + irritable</td></tr>
<tr><td>☐</td><td>informed + team builder + integrative</td><td>☐</td><td>self-interested + loner + asocial</td></tr>
<tr><th colspan="2">How a <u>follower</u> should definitely be ...</th><th colspan="2">How a <u>follower</u> should not be at all ...</th></tr>
<tr><td>☐</td><td>hardworking + productive + goes above and beyond</td><td>☐</td><td>easily influenced + follows trends + soft spoken</td></tr>
<tr><td>☐</td><td>excited + outgoing + happy</td><td>☐</td><td>arrogant + rude + bad-tempered</td></tr>
<tr><td>☐</td><td>loyal + reliable + team player</td><td>☐</td><td>uneducated + slow + inexperienced</td></tr>
</table></td></tr>
<tr><td>1.6</td><td>Please wait</td><td>Thank you! Please wait until we have processed your selection.</td></tr>
<tr><td colspan="3">Part II Vignettes</td></tr>
<tr><td>2.0</td><td>Instruction vignettes</td><td> Great - your personal profile has been created.
In the next part of the experiment, you will be presented the anonymized personal profiles of your co-players. Please indicate for each profile how much you would like to engage in job sharing with this person. Please use the scale below each profile to indicate your preferences.
Please note: Consider every single profile carefully and provide ratings for each profile presented to you! </td></tr>
<tr><td>2.1 – 2.6</td><td>Vignettes</td><td> [See example]
Please indicate the extent to which you would like to engage in job sharing with this person
- 1 - not all
- 7- very much </td></tr>
<tr><td>2.7</td><td>Please wait</td><td>Thank you! Now, please wait until the algorithm has assigned you a partner.</td></tr>
<tr><td>2.8</td><td>Partner assignment</td><td> Thank you. You have been assigned a partner with the following profile:
[vignette] </td></tr>
</table>

**Part III Cooperation Task**		
3.0	Task instruction	You have now reached the last part of the experiment. Here, you and your assigned partner will complete the job sharing task together.<br>Please read the task instructions carefully.<br><u>Background</u><br>Please imagine that you and your partner share a leadership position in an industrial company. Each of you occupies the position for half the working week (2.5 days). For the last weeks, the two have you have also been sharing the lead for a prestigious digitalization project. To expand the project team, you want to hire an IT specialist who will report to both of you.<br>From a large number of applications, the HR department has selected the three best candidates. Both you and your partner have received important information about each of the three candidates. However, since you spoke to different HR representatives, you and your partner are provided different sets of information about the candidates. In order to arrive at a qualified hiring decision, you need to share and discuss your information about all of the applicants.<br><u>Your task</u><br>Your task consists in deciding who of the three proposed candidates is most qualified for the job and therefore should be hired. Since you and your partner possess different information about the candidates' qualifications, it is important that you discuss every information thoroughly and come to a shared decision.
3.1	Tables of information	Please read your set of information carefully. You will be provided the same overview on the following page, so you don't have to learn the facts by hard. Your partner has different information.<br>Please discuss with your partner whom of the candidates you would hire. Please pay particular attention to each candidate's professional and personal suitability. To communicate with your partner, please use the chat window on the next page.<br>When you have decided on a candidate, log in the name of the candidate whom you would hire in the boxes on the last page.<br>Once you are ready, click continue.
3.2	Please wait	Please wait until your partner is ready.
3.3	Discussion via chat window	[*information overview*]<br>[*chat window*]<br>When you have reached a joint decision, please click "Next".
3.4	Decision	*Which candidate do you choose to hire?*<br>- *Candidate A*<br>- *Candidate B*<br>- *Candidate C*<br>- *We have not reached a decision*
3.5	If applicable, notification about prize	[*in case of a win*]<br>Congratulations! You have logged in the correct answer and have been drawn from all winning couples. Both you and your partner will receive an extra 10 € on top of your payout.

3.6	Debriefing	Thank you for your participation. You have successfully completed the study.<br>Lastly, we would like to briefly explain the background and objectives of the study. This study sets out to investigate how people's unconscious expectations of good or bad employees and leaders influencer their choices of a job sharing partner. To do this, we first tested which of your own images of very good or very bad employees and leaders you chose to express to others (round 1). In round 2, you were randomly shown a series of fictitious personal profiles that communicated different views of particularly good or bad leaders and employees. We then measured which of these profiles you found most attractive and how your own expressed preferences affected your attraction to unknown job-sharing partners.<br>In case you are interested in the results of the study or have general questions or comments, please feel free to contact us at [email address].

Appendix B. Sample Personal Profile / Sample Vignette for the Espoused Dimensions *Inspirational* and *Insubordination*

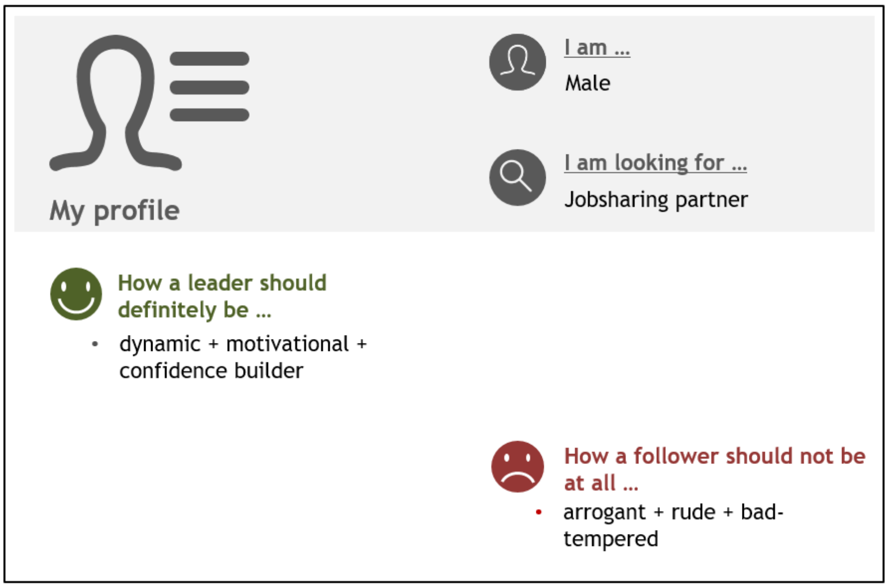

Appendix C. Pairwise Contrasts for Levels of Congruence Variables (Coded Categorically)

	**Differences between numbers of matches**														
	0 vs. 1					0 vs. 2					1 vs. 2				
Predictor	*Estimate*	*SE*	*df*	*t*	*p*	*Estimate*	*SE*	*df*	*t*	*p*	*Estimate*	*SE*	*df*	*t*	*p*
Prototype Congruence	-.538***	.093	1513	-5.762	<.001	-1.056***	.173	1513	-6.091	<.001	-.518*	.163	1504	-3.180	.004
Antiprototype Congruence	-.308**	.104	1140	-2.966	.009	-.408	.262	1507	-1.560	.263	-.100	.257	1485	-.390	.919
Leader Prototype Congruence	-.332**	.109	1513	-3.052	.006	-.240	.923	1419	-.260	.963	.092	.921	1403	.100	.994
Follower Prototype Congruence	-.368**	.107	1484	-3.460	.001	*N/A*	*N/A*	*N/A*	*N/A*	*N/A*	*N/A*	*N/A*	*N/A*	*N/A*	*N/A*

Note. Number of matches counts how often self and other matched on espoused ILT/IFT prototypes, antiprototypes, and leader/follower prototypes, respectively. Marginal means are only reported for significant predictors. *N/A* indicates that not enough observations per level were available to calculate contrasts.

*** $p < .001$, ** $p < .01$, * $p < .05$.
